# Beyond the Classical View: Early Emergence of Class Switch Recombination-Associated Molecular Features During B-Cell Development in the Bone Marrow

**DOI:** 10.3390/ijms27188074

**Published:** 2026-09-11

**Authors:** Kawtar Hanefioui, Mohamad Omar Ashi, Fadila Guessous

**Affiliations:** 1 Faculty of Medicine, Mohammed VI University of Health Sciences, Casablanca 82403, Morocco; khanefioui@um6ss.ma; 2 Laboratory of Oncopathology, Biology and Environment of Cancer, Mohammed VI Center for Research and Innovation, Rabat 10112, Morocco; 3 Departments of Medical Pathology and Biology, Gustave Roussy Institute, 94805 Villejuif, France; omarashi@live.com

**Keywords:** B-cell, bone marrow, class switch recombination, switch transcription, *IgH* enhancers

## Abstract

Class switch recombination (CSR) is a fundamental mechanism of humoral immunity that enables activated B lymphocytes to switch from the expression of IgM to other immunoglobulin isotypes, such as IgG, IgA, or IgE, thereby changing the effector functions while preserving antigen specificity. Traditionally, CSR has been considered a late event in B-cell differentiation, occurring predominantly in germinal centers following antigen encounter and activation-induced cytidine deaminase (AID) expression. In this classical model, B-cell development in the bone marrow is largely separated from antigen-dependent antibody diversification in peripheral lymphoid organs. However, transcriptomic, epigenomic, and functional studies suggest that several molecular components and regulatory features associated with CSR may already emerge during early stages of B-cell ontogeny. Evidence from mouse models and molecular studies indicates that immature and transitional B cells can express low levels of AID, while transcriptional and regulatory features linked to CSR may already be established before antigen-driven B-cell activation. In this review, we first summarize the classical process of CSR, including the molecular events and requirements necessary for its activation in mature B cells. We then discuss evidence indicating that components and regulatory features associated with CSR can emerge during early B-cell development. Furthermore, we examine how transcriptional regulation and enhancer activity within the IgH locus may contribute to the establishment of these features throughout ontogeny. Finally, we discuss the potential biological implications of early CSR-associated molecular activity for immune repertoire formation, central tolerance, genomic integrity, and B-cell malignancies.

## 1. Introduction

CSR involves a DNA recombination process that replaces the Cμ constant gene, which encodes the constant region of the IgM heavy chain, with another downstream constant gene within the immunoglobulin heavy-chain (IgH) locus, leading to the expression of a different antibody isotype. The V(D)J variable region responsible for antigen specificity remains intact [1,2]. Following antigen encounter in secondary lymphoid organs, activated B lymphocytes can undergo CSR in response to signals including CD40-CD40L interactions, Toll-like receptor (TLR) engagement, and cytokine stimulation, which regulate the expression of AID [1,2]. AID initiates the molecular events leading to CSR by deaminating cytosine residues within immunoglobulin switch (S) regions. The resulting DNA lesions are processed by DNA repair pathways, ultimately allowing recombination between S regions and replacement of the expressed constant region [1,3].

The classical view therefore places antibody diversification after the completion of antigen-independent B-cell development in the bone marrow. However, this temporal separation is not absolute. Several studies have detected molecular features associated with CSR during earlier stages of B-cell development, including AID expression and regulatory changes involving the IgH locus [4,5]. In parallel, the activity of regulatory elements such as the 3′ regulatory region (3′RR) and CCCTC-binding factor (CTCF) has been implicated in the control of IgH locus transcription and accessibility [6,7]. These observations raise the possibility that molecular conditions associated with CSR may be established progressively during B-cell ontogeny, before conventional antigen-driven activation.

Importantly, the detection of individual molecular features associated with CSR does not necessarily demonstrate that productive recombination has occurred. CSR-associated molecular features refer to the presence of individual components or regulatory features linked to CSR, such as AID expression, germline transcription, or increased accessibility of the IgH locus, without direct evidence of recombination. CSR competence refers to the ability of developing B cells to undergo class switching following appropriate stimulation, particularly in experimental settings in which CSR is induced ex vivo. Early CSR priming refers to the establishment of molecular and chromatin features that prepare the IgH locus for subsequent recombination, without necessarily resulting in CSR at that developmental stage. The term early CSR activity is used more broadly to describe CSR-associated molecular activity detected during early B-cell development and does not, by itself, imply that completed recombination has occurred in vivo. Completed CSR is reserved for studies providing direct evidence of recombination products, such as circle transcripts, post-switch transcripts, or genomic Sμ-to-Sx junctions. These categories are applied consistently throughout this review and are used to classify the studies summarised.

## 2. Overview of B-Cell Development and Associated Molecular Events

**Figure 1:**
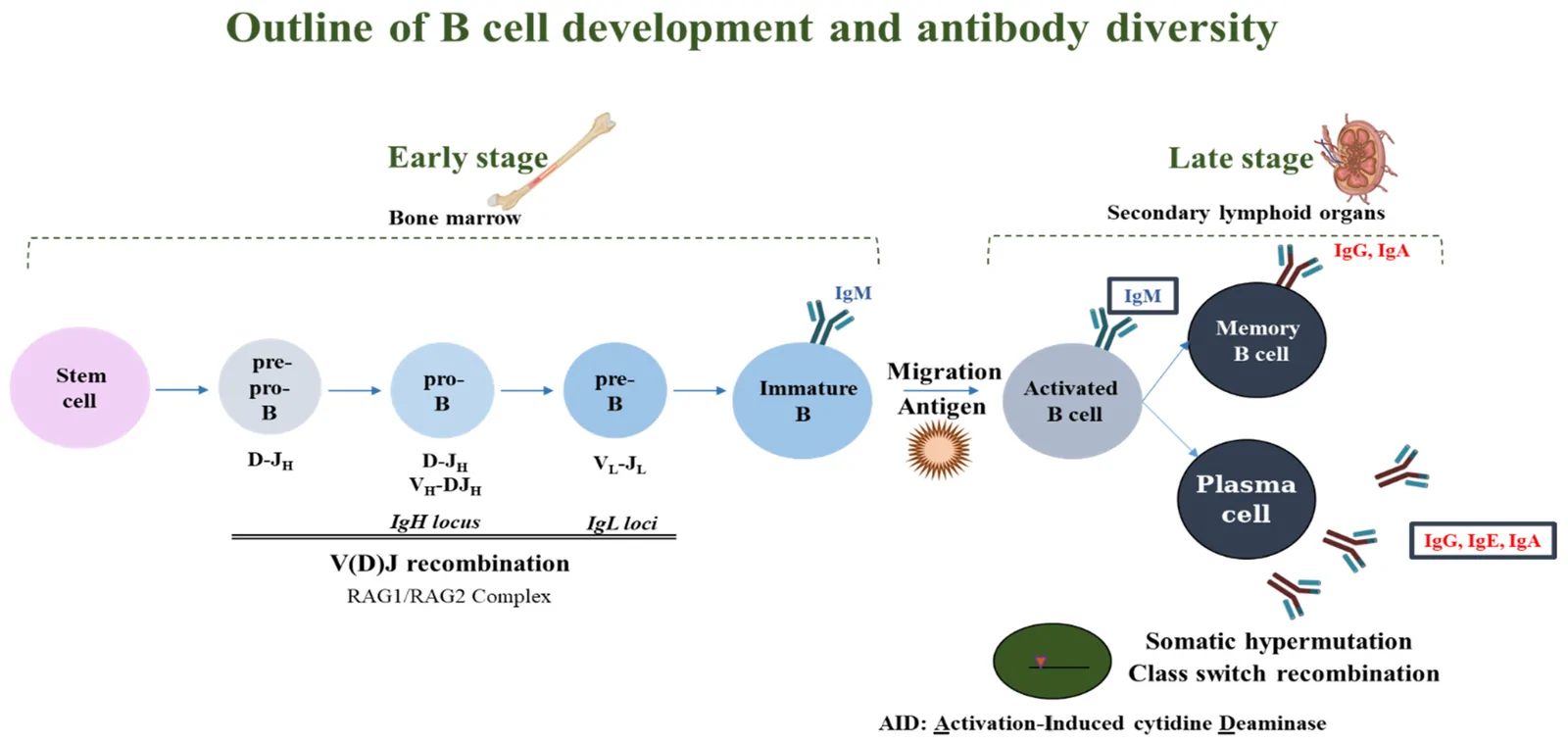
Outline of B-cell development and antibody diversity. The triangle represents the AID enzyme.

B-cell development is a tightly regulated process that ensures the generation of a diverse yet self-tolerant antibody repertoire. It can be broadly divided into antigen-independent development in primary lymphoid organs and antigen-dependent maturation in peripheral lymphoid tissues (Figure 1). These two phases are characterized by distinct cellular, molecular, and functional events that together establish the basis of humoral immunity.

Antigen-independent development in the bone marrow (V(D)J recombination by RAG1/RAG2, which assembles a functional IgM B-cell receptor) is followed, after migration and antigen encounter, by antigen-dependent maturation in secondary lymphoid organs, where somatic hypermutation (SHM) and class switch recombination (CSR), both mediated by AID, drive affinity maturation and isotype diversity.

The antigen-independent phase occurs primarily in the bone marrow, where hematopoietic progenitors undergo sequential differentiation into pro-B, pre-B, and immature B cells. During this process, B cells rearrange their immunoglobulin heavy-chain (IgH) and light-chain loci through V(D)J recombination mediated by RAG1 and RAG2 [8]. This generates a functional B-cell receptor (BCR), expressed as IgM isotype. Immature B cells then undergo central tolerance checkpoints to eliminate or inactivate autoreactive clones before exiting the bone marrow [8].

After leaving the bone marrow, transitional B cells complete their maturation in peripheral lymphoid organs, contributing to the naïve B-cell pool. These cells co-express IgM and IgD and circulate until antigen encounter initiates activation and differentiation. The antigen-dependent phase is triggered by antigen recognition through the BCR, together with co-stimulatory signals from T helper cells or innate immune receptors. Activated B cells may differentiate into short-lived plasma cells or enter germinal center reactions in secondary lymphoid organs, which represent the central sites of antibody affinity maturation and isotype diversity [9,10]. Within germinal centers, B cells undergo two fundamental processes, somatic hypermutation (SHM) and class switch recombination (CSR), both dependent on activation-induced cytidine deaminase (AID), encoded by the Aicda gene [3,11,12]. SHM introduces point mutations into immunoglobulin variable regions, enabling affinity-based selection of high-affinity clones. CSR modifies the constant region of the IgH locus, allowing switching from IgM/IgD to IgG, IgA, or IgE without altering antigen specificity [9,10]. CSR and SHM are induced by integrated signaling pathways including CD40-CD40L interactions, Toll-like receptor signaling, and cytokines such as IL-4, IFN-γ, and TGF-β [13,14]. These signals converge to regulate AID expression and activation of the immunoglobulin remodeling program.

Beyond enzymatic regulation, CSR requires chromatin accessibility at the IgH locus. Germline transcription of switch regions is essential for AID targeting, as it exposes single-stranded DNA substrates. This transcription is regulated by cis-elements within the 3′ immunoglobulin heavy-chain regulatory region (3′RR), a super-enhancer controlling locus accessibility and transcriptional competence [15,16,17].

Together, these processes define the classical model of B-cell development, in which antigen-independent maturation in the bone marrow is strictly separated from antigen-driven antibody diversity in germinal centers. This paradigm has long structured our understanding of humoral immunity.

However, emerging evidence suggests that components of this regulatory network may also be active earlier during B-cell ontogeny, challenging the strict temporal separation between development and class switching, thus motivating a reassessment of CSR timing.

## 3. Molecular Machinery of CSR in Mature B Cells

CSR is a DNA recombination process that occurs predominantly in activated B cells, enabling the modification of the IgH constant region while preserving antigen specificity. Through CSR, the Cμ constant region is replaced by downstream constant region genes, allowing the production of different antibody isotypes, including IgG, IgA, or IgE. Although CSR can be induced in both T-dependent and, to a more limited extent, T-independent immune responses, it is most efficiently triggered in the context of T cell help. Defects in this process lead to humoral immunodeficiency syndromes characterized by elevated or normal IgM levels and impaired production of switched isotypes, resulting in increased susceptibility to recurrent infections [18,19].

**Figure 2:**
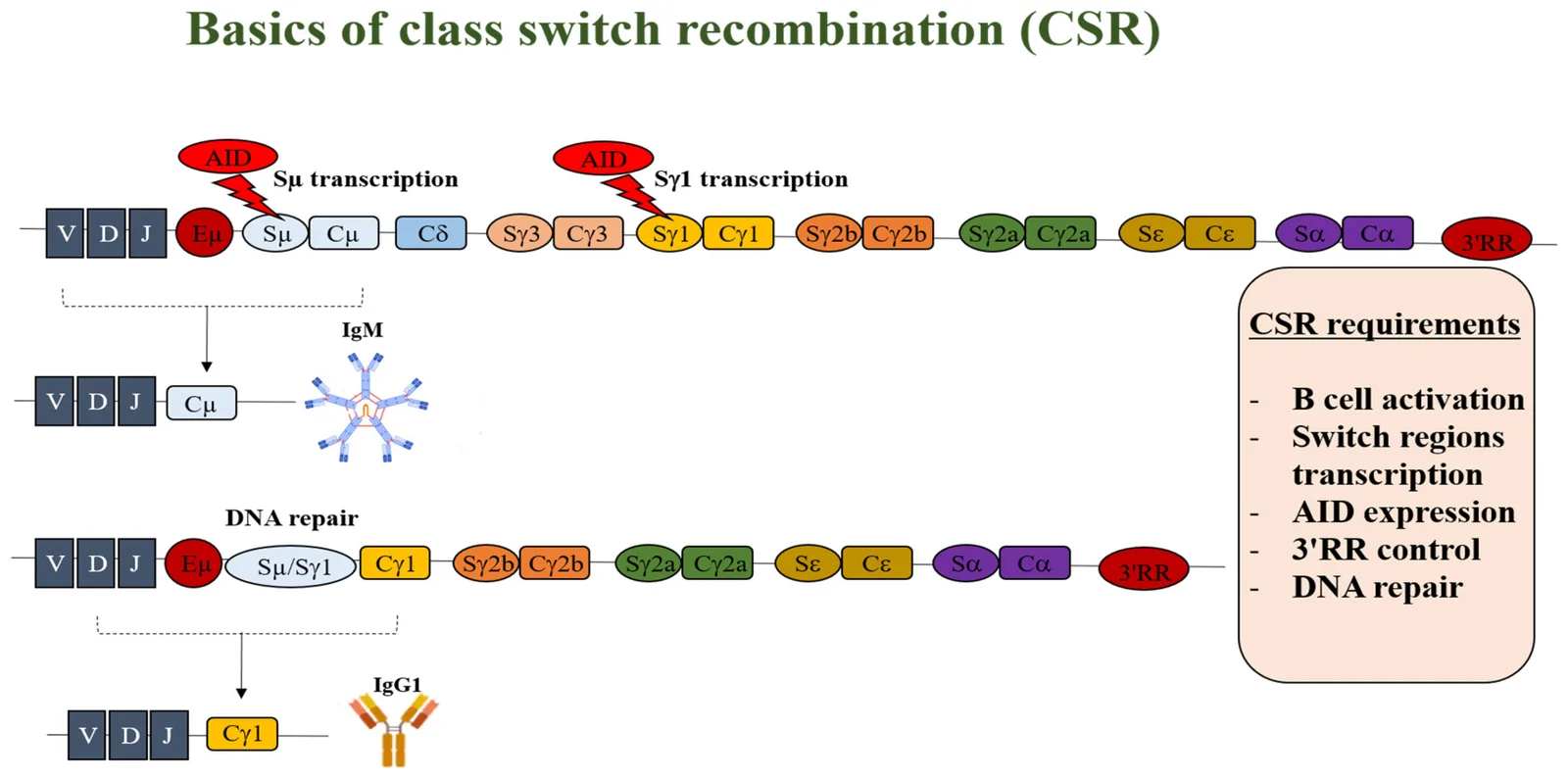
Basics of class switch recombination (CSR) at the mouse immunoglobulin heavy-chain locus.

At the molecular level, CSR occurs within repetitive switch (S) regions located upstream of immunoglobulin constant region exons, with the exception of Cδ. The process involves recombination between the donor Sμ region and a downstream acceptor S region (Figure 2), resulting in deletion of the intervening genomic DNA as an excised circular episome [19,20].

Germline transcription of the donor (Sμ) and an acceptor (e.g., Sγ1) switch region renders them accessible to AID; AID-initiated deamination and downstream DNA repair generate double-strand breaks whose joining replaces the constant region (here IgM to IgG1) while preserving the V(D)J exon. CSR requires B-cell activation, switch-region transcription, AID expression, 3′RR control, and DNA repair.

CSR is orchestrated through a tightly regulated sequence of molecular events that ensure accessibility, targeting, and repair of switch regions. A first essential step is antigen-dependent B-cell activation, which integrates signals from the B-cell receptor together with co-stimulatory cues provided by T cells or innate immune receptors. These include CD40–CD40L interactions, Toll-like receptor (TLR) signaling, and cytokine-mediated cues such as IL-4, IFN-γ, and TGF-β, which collectively induce transcriptional programs required for CSR initiation [13,14].

A key prerequisite for CSR is the transcriptional activation of switch regions, referred to as germline or “pre-switch” transcription. This transcriptional activity renders S regions accessible to AID. Transcription through S regions is generally bidirectional and orientation-dependent, facilitating the formation of RNA:DNA hybrids (R-loops) that expose single-stranded DNA substrates for AID targeting. RNA processing and degradation of germline transcripts by the RNA exosome further contribute to the generation of accessible DNA substrates, while replication protein A (RPA) stabilizes single-stranded DNA intermediates to promote efficient AID activity [21,22,23,24]. Germline transcription is constitutive at the Sμ region but inducible at downstream acceptor S regions following B-cell activation [1,25,26].

The central enzyme of CSR, AID (encoded by *Aicda* gene), is induced upon B-cell activation through signaling pathways downstream of CD40 engagement, TLR stimulation, and cytokine receptors. AID catalyzes the deamination of cytosine residues into uracils within transcribed switch regions, generating U:G mismatches that are subsequently processed by base excision repair (BER) and mismatch repair (MMR) pathways. These repair processes convert initial lesions into DNA strand breaks that are essential intermediates for recombination. AID activity is not restricted to immunoglobulin loci: genome-wide translocation sequencing has mapped AID-dependent breakpoints throughout the B-cell genome, concentrated at transcribed regions and at convergently transcribed intragenic super-enhancers, from which translocations to IgH arise [27,28]. Strict control of AID expression and targeting is therefore required to limit genomic instability and malignant transformation [29].

The completion of CSR requires the formation and joining of DNA double-strand breaks (DSBs) within donor and acceptor switch regions. These DSBs arise when closely spaced single-strand breaks are generated on opposing DNA strands. Repair is predominantly mediated by the classical non-homologous end joining (c-NHEJ) pathway, which ligates the broken DNA ends and excises the intervening genomic segment, thereby completing isotype switching [2,22]. c-NHEJ is not the only available route: in the absence of core c-NHEJ factors, an alternative end-joining (A-EJ) pathway supports a substantial fraction of switching, producing junctions enriched in microhomology and an increased frequency of translocations [30]. Pathway choice is regulated, in particular by 53BP1, which protects switch-region ends against resection and thereby favours c-NHEJ [31]. Because these repair factors are differentially expressed across B-cell development, the way a switch-region break is resolved may itself differ between precursor and mature B cells.

Finally, CSR efficiency is critically regulated by the 3′ regulatory region (3′RR), a super-enhancer complex located downstream of the immunoglobulin heavy chain locus. The 3′RR comprises multiple enhancer elements, including hs3a, hs1.2, hs3b, and hs4, which progressively acquire activating chromatin modifications during B-cell differentiation. These elements act cooperatively to promote germline transcription and CSR, in part through enhancer RNA (eRNA) production and long-range chromatin interactions. Genetic disruption of the 3′RR results in severe defects in class switching, underscoring its essential role in regulating immunoglobulin diversity [15,16,17,32].

Overall, CSR relies on the coordinated succession of essential cellular and molecular events. B-cell activation provides the signals that initiate the CSR program, followed by AID expression and targeting, germline transcription of switch regions, and the processing and repair of AID-induced DNA lesions to enable recombination. In parallel, the 3′ regulatory region (3′RR) contributes to the transcriptional regulation required for efficient switching. Together, these coordinated events constitute essential prerequisites for efficient CSR and ensure its proper initiation, execution, and completion.

## 4. Early Establishment of Molecular Features Associated with CSR During B-Cell Development

Although CSR is classically restricted to antigen-experienced mature B cells within secondary lymphoid organs, accumulating evidence suggests that key components of the CSR machinery may already be expressed and functionally engaged during early B-cell development. This view challenges the strict dichotomy between developmental B-cell programming and activation-induced immunoglobulin diversity, and raises the possibility that CSR competence is progressively established prior to full antigen-dependent activation. Evidence for this early establishment comes from the detection of several key molecular features associated with CSR during antigen-independent B-cell development, summarised in Table 1.

### 4.1. AID Expression Precedes Antigen Encounter

In the mouse, Aicda expression has been detected in pre-B and immature B cells of the bone marrow, with its induction depending on B-cell receptor and Toll-like receptor signalling rather than on T-cell help [4]. Single-cell analyses of the mouse bone-marrow B lineage are consistent with a low-level, developmentally distributed Aicda programme rather than a sharply delimited AID-positive stage [6]. Given its mutagenic potential, AID is normally regulated at transcriptional, post-transcriptional and post-translational levels [13,14].

AID is also detected in human bone-marrow immature B cells, but the associated phenotype differs. AID knockdown in B cells developing in humanised mice caused a failure to remove autoreactive clones, indicating a requirement for AID in central B-cell tolerance [5]. The mechanism proposed operates through receptor editing and apoptotic deletion, processes that require AID but are at least partly independent of CSR, and AID-deficient mice do not reproduce the human tolerance defect in the same manner [33]. Mouse and human observations should therefore not be considered interchangeable: the available murine evidence includes early AID expression and CSR-related activity, whereas the human evidence discussed here primarily concerns the role of AID in central B-cell tolerance. This divergence limits the generality of the model proposed here. Early AID expression may be a conserved feature of B-cell development whose downstream use differs between species, being directed partly towards switching in the mouse and primarily towards repertoire editing in humans. Whether this reflects a genuine species difference or the different tissues and experimental approaches accessible in each system is currently unresolved, and conclusions drawn from murine models should therefore not be transposed directly to human B-cell development.

### 4.2. Germline Transcription Is Restrained Rather than Absent

AID expression alone is insufficient, since CSR also requires transcriptionally active switch regions [1]. Although the 3′RR plays an essential role in the transcriptional regulation required for CSR, its activity is tightly restricted during the early stages of B-cell development. This restriction involves the CTCF-binding element (CBE) 5′hs1RI, located downstream of the Cα constant gene. The CTCF factor binds to this site and acts as a barrier by limiting enhancer–promoter interactions, thereby contributing to the restriction of premature germline transcription of switch regions [34,35,36,37].

**Figure 3:**
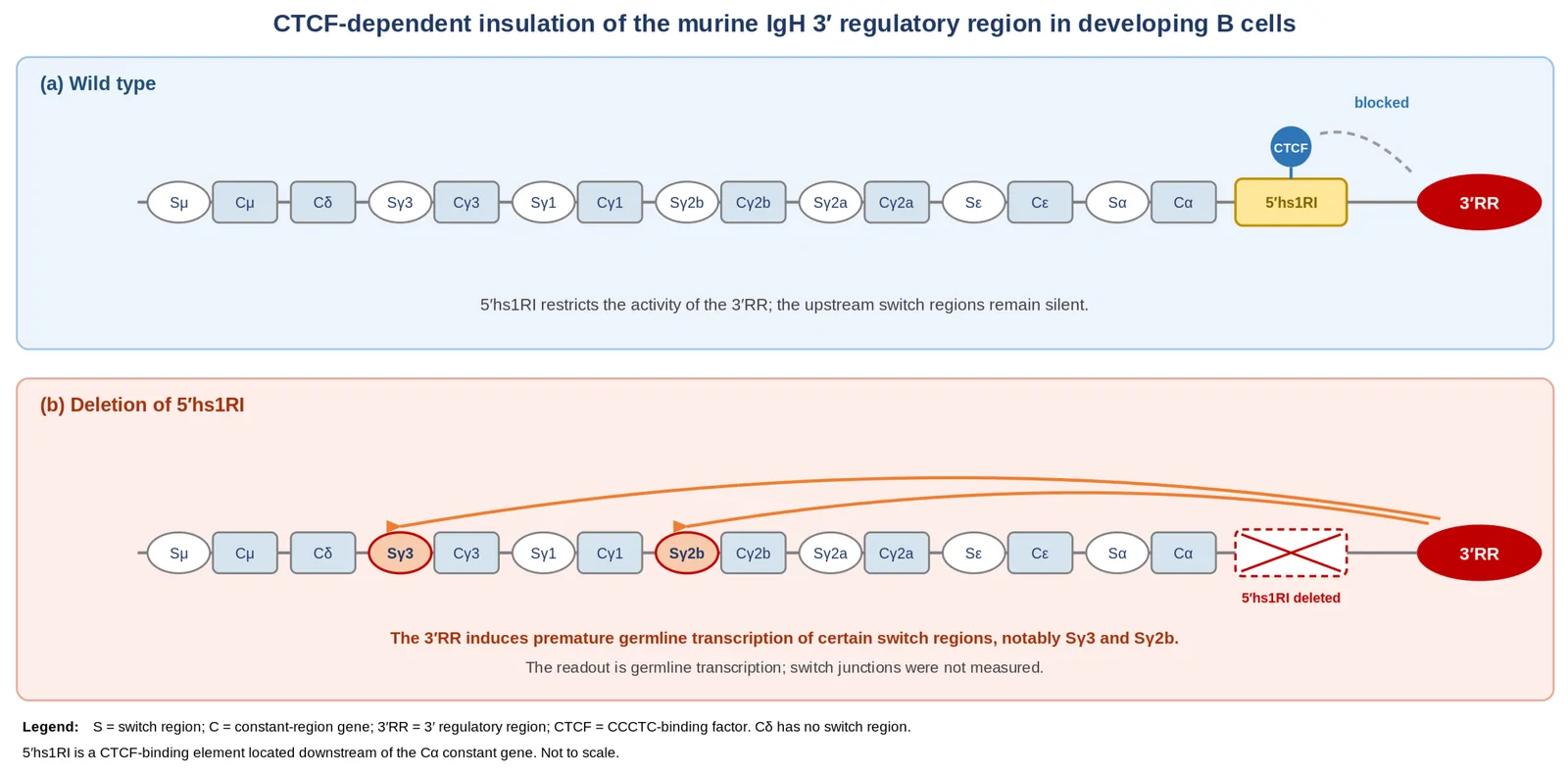
CTCF-dependent insulation of the murine IgH 3′ regulatory region (3′RR) in developing B cells.

In a mouse model carrying a 5′hs1RI deletion, removal of this element allows the 3′RR to induce early activation of germline promoters and premature transcription of certain switch regions, notably Sγ3 and Sγ2b, in pro-B cells as well as resting splenic B cells (Figure 3) [7]. This effect affects a subset of switch regions rather than the locus as a whole. However, the readout corresponds to germline transcription rather than switch junctions; therefore, the experiment demonstrates that the locus is primed for CSR but does not establish that recombination has actually occurred.

CTCF acts as a barrier that limits 3′RR-mediated activation of germline promoters during early B-cell development. Disruption of this insulation leads to premature activation and transcription of specific switch regions, notably Sγ3 at the pro-B-cell stage and Sγ2b at the pre-B-cell stage, whereas other switch regions show little or no detectable activation under these conditions. In the presence of CTCF binding (a), the switch regions shown in the diagram remain transcriptionally silent. Deletion of the CTCF-binding site prevents CTCF binding and removes this insulating barrier (b), allowing the 3′RR to activate germline transcription at specific switch regions.

### 4.3. The 3′RR Is Required for the Switching Capacity of Pro-B Cells

Further evidence for the involvement of the 3′RR during early B-cell development comes from studies showing that its deletion markedly impairs switch transcription and CSR in pro-B cells. Deletion of the 3′RR results in a profound reduction in switch transcripts, including Sμ, Sγ3, Sγ2b, Sγ1, and Sε, and a near-complete loss of CSR in early B-cell precursors [38]. Interestingly, among the enhancer modules comprising the 3′RR, only hs4 displays detectable activity at this developmental stage, as indicated by the detection of its eRNA, whereas eRNAs from the other enhancers remain at low or undetectable levels. This stage-specific enhancer activity may contribute to the low but detectable levels of switch-region transcription observed in developing B cells [38]. The experimental context should be kept in mind when interpreting this result: CSR was assessed after ex vivo stimulation of cultured pro-B cells, and not in bone marrow or secondary lymphoid organs in situ. The study therefore establishes that the 3′RR is required for the switching capacity of pro-B cells, that is, for CSR competence as defined above, rather than demonstrating that CSR occurs in pro-B cells during unperturbed development.

### 4.4. Evidence of Completed Recombination

Two mouse studies go beyond the detection of AID. Circle transcripts, produced from the excised episome and therefore serving as short-lived markers of recombination that has just occurred, have been detected together with post-switch transcripts in sorted bone-marrow B-cell subsets [4]. Immature B cells have been reported to switch preferentially to IgE, with an increased proportion of direct Sμ-to-Sε recombination compared with the sequential route used by mature B cells [39]. Since acceptor choice depends on which germline promoters the 3′RR can reach, this bias indicates that the developing locus is configured differently rather than simply being less active. The same group later described B-lineage cells developing in the intestinal lamina propria of postnatal mice, raising the possibility that part of this switching occurs outside the bone marrow [40].

**Table 1:** Primary studies discussed in Section 3, listing for each the species, the developmental B-cell subset analysed, the experimental method, and the level of evidence the result provides according to the categories defined in the Introduction. Completed CSR is the only category that demonstrates recombination; the others describe molecular features, priming or competence.

Study	Species	B-Cell Subset Analysed	Method	Level of Evidence
Han et al. [4]	Mouse	Bone-marrow pre-B and immature B cells	Aicda RT-PCR; circle transcripts; post-switch transcripts	Completed CSR
Wesemann et al. [39]	Mouse	Immature B cells, stimulated ex vivo	Surface IgE staining; direct versus sequential Sμ-to-Sε junctions	Completed CSR, after stimulation
Lee et al. [6]	Mouse	Bone-marrow B-lineage developmental continuum	Single-cell RNA sequencing	CSR-associated molecular feature (Aicda transcripts)
Cantaert et al. [5]	Human	Bone-marrow immature B cells; humanised mice	AID protein detection; shRNA knockdown; autoreactivity of cloned antibodies	CSR-associated molecular feature; tolerance phenotype, no CSR readout
Braikia et al. [7]	Mouse	Pro-B cells and resting splenic B cells (Δ5′hs1RI)	Germline transcript quantification by RT-qPCR	Early CSR priming
Dauba et al. [38]	Mouse	Pro-B cells enriched in culture (Δ3′RR)	Switch transcript quantification; CSR scored after ex vivo stimulation	CSR competence

### 4.5. Limits of the Available Evidence

Two methodological points limit how far these observations can be taken. First, the murine bone-marrow B220+IgM+ compartment is heterogeneous: it contains recirculating mature B cells alongside newly generated immature cells, and recirculating mature cells are precisely those expected to carry AID and switch products [8]. The two populations can be separated, since immature B cells are IgM+ IgD (low/negative) and CD23 (negative) and express high levels of CD93, whereas recirculating mature B cells are IgM+ IgD (high) CD23 (positive) and CD93 (negative); pre-B cells, which are surface IgM (negative), have no recirculating mature counterpart at all. Conclusions about early CSR therefore depend on whether a given study resolved these subsets, and reports that included pre-B cells are less exposed to this ambiguity than those relying on B220 and IgM alone [4]. Second, where CSR has been scored after ex vivo stimulation of sorted precursors, the experiment establishes CSR competence rather than an event occurring during unperturbed development [38]. Neither point contradicts the presence of CSR-associated features early in development, but both constrain what may be concluded from it, and both could be resolved by lineage tracing combined with direct detection of recombination products.

## 5. Mechanistic Interpretation and Biological Significance

Whether the molecular features described above culminate in productive recombination or reflect developmental priming of the IgH locus remains unresolved. Three interpretations, which are not mutually exclusive, deserve consideration.

The first is that early CSR-associated activity represents a preparatory state. Progressive chromatin remodelling at the IgH locus during ontogeny may establish accessibility patterns that are subsequently exploited in germinal centre reactions, without themselves leading to recombination. This is consistent with models of lineage specification in which enhancer activation precedes full transcriptional competence [17,41,42]. The loop-extrusion framework gives this interpretation a physical basis: developmental insulation constrains the reach of the 3′RR rather than abolishing its activity, which accounts for the restricted set of switch regions affected when a single CTCF-binding element is removed [7,43,44].

A second possibility is that limited CSR-related activity contributes to central B-cell tolerance. This rests principally on human data, where AID expression in immature B cells participates in the elimination or editing of autoreactive clones through receptor editing and apoptosis rather than through switching itself [5,33]. Extending this interpretation to the mouse is not currently justified.

A third possibility is that early activity is a genomically hazardous by-product. AID is intrinsically mutagenic, and low levels of aberrant activity can generate off-target mutations and chromosomal rearrangements [27,29]. Since the repair pathways available to a switch-region break differ across development, a lesion arising in a precursor may not be resolved as faithfully as in a mature cell [30]. Consistent with this, inappropriate AID expression has been implicated in the pathogenesis of precursor B-cell acute lymphoblastic leukaemia [45], and the DNA damage response provides an additional layer of control over the switching reaction [46].

Distinguishing between these possibilities will require lineage tracing that assigns a recombination event to a cell of known developmental history, single-cell approaches combining Aicda expression with switch-region accessibility, and direct detection of recombination products in stringently sorted subsets [6].

## 6. Conclusions

The evidence reviewed here is not uniform in weight, and separating its levels changes what can be concluded. The mouse, circle and post-switch transcripts in sorted bone-marrow subsets, and an IgE-biased switching preference in immature B cells, indicate that recombination can be completed before the germinal centre [4,39]. The enhancer- and insulator-dependent studies establish that the locus is primed and that precursors are competent when stimulated, but were scored on germline transcripts or after ex vivo stimulation, and therefore do not by themselves place a recombination event in the bone marrow in vivo [7,38]. In humans, the early AID requirement is best supported as a tolerance mechanism [5,33].

These findings qualify the classical model rather than overturn it. CSR competence appears to be assembled progressively during ontogeny and held under active restraint, rather than acquired abruptly upon antigen encounter. How frequently early recombination occurs in unperturbed animals, whether it contributes to the peripheral repertoire, and whether the mouse and human situations are mechanistically equivalent remain open questions whose resolution bears on repertoire formation, self-tolerance and susceptibility to malignant transformation.

## Data Availability

No new data were created or analyzed in this study. Data sharing is not applicable to this article.

## Abbreviations

The following abbreviations are used in this manuscript:

CSR	Class switch recombination
3′RR	3′ regulatory region
IgH	Immunoglobulin heavy chain
AID	Activation-induced cytidine deaminase
A-EJ	Alternative end joining
BCR	B-cell receptor
BER	Base excision repair
C-NHEJ	Classical non-homologous end joining
CTCF	CCCTC-binding factor
DSB	Double-strand break
eRNA	Enhancer RNA
MMR	Mismatch repair
RAG	Recombination-activating gene
SHM	Somatic hypermutation
TLR	Toll-like receptor
